# Cellular and molecular roles of reactive oxygen species in wound healing

**DOI:** 10.1038/s42003-024-07219-w

**Published:** 2024-11-19

**Authors:** Matthew Hunt, Monica Torres, Etty Bachar-Wikstrom, Jakob D. Wikstrom

**Affiliations:** 1 Dermatology and Venereology Division, Department of Medicine (Solna), Karolinska Institutet, Stockholm, Sweden; 2 Dermato-Venereology Clinic, Karolinska University Hospital, Stockholm, Sweden

**Keywords:** Molecular medicine, Medical research

## Abstract

Wound healing is a highly coordinated spatiotemporal sequence of events involving several cell types and tissues. The process of wound healing requires strict regulation, and its disruption can lead to the formation of chronic wounds, which can have a significant impact on an individual’s health as well as on worldwide healthcare expenditure. One essential aspect within the cellular and molecular regulation of wound healing pathogenesis is that of reactive oxygen species (ROS) and oxidative stress. Wounding significantly elevates levels of ROS, and an array of various reactive species are involved in modulating the wound healing process, such as through antimicrobial activities and signal transduction. However, as in many pathologies, ROS play an antagonistic pleiotropic role in wound healing, and can be a pathogenic factor in the formation of chronic wounds. Whilst advances in targeting ROS and oxidative stress have led to the development of novel pre-clinical therapeutic methods, due to the complex nature of ROS in wound healing, gaps in knowledge remain concerning the specific cellular and molecular functions of ROS in wound healing. In this review, we highlight current knowledge of these functions, and discuss the potential future direction of new studies, and how these pathways may be targeted in future pre-clinical studies.

## Abstract (web summary)

This review highlights the cellular and molecular mechanisms in which ROS are involved in normal wound healing and chronic wound pathogenesis, as well as recent advances in therapeutic methods.

## Introduction

Dermatological would healing is the tightly coordinated response of restoring skin tissue integrity and homeostasis following damage. Involving numerous immune and non-immune cell types as well as associated cytokines, growth factors, and extracellular components – in a healthy, acute response – orderly wound healing consists of the four consecutive stages of haemostasis, inflammation, proliferation, and tissue remodelling^1,2^. Briefly, during the haemostasis phase, platelets are recruited or leak out of damaged vasculature and with the simultaneous activation of the coagulation cascade and formation of fibrin fibres, form a clot at the wound site^3^. Next, during inflammation, various immune cells migrate to the wound site and utilise phagocytotic effects to protect against infection. Additionally, these immune cells also release pro-inflammatory cytokines and growth factors to induce the activation of fibroblasts, keratinocytes, and endothelial cells, as well as prepare the wound bed for the formation of granulation tissue^4,5^. During the proliferation phase, granulation tissue is formed and damaged tissue is replaced. At the remodelling phase, connective tissue, replacement epithelium, and scar tissue are all formed^6^.

**Fig. 1:**
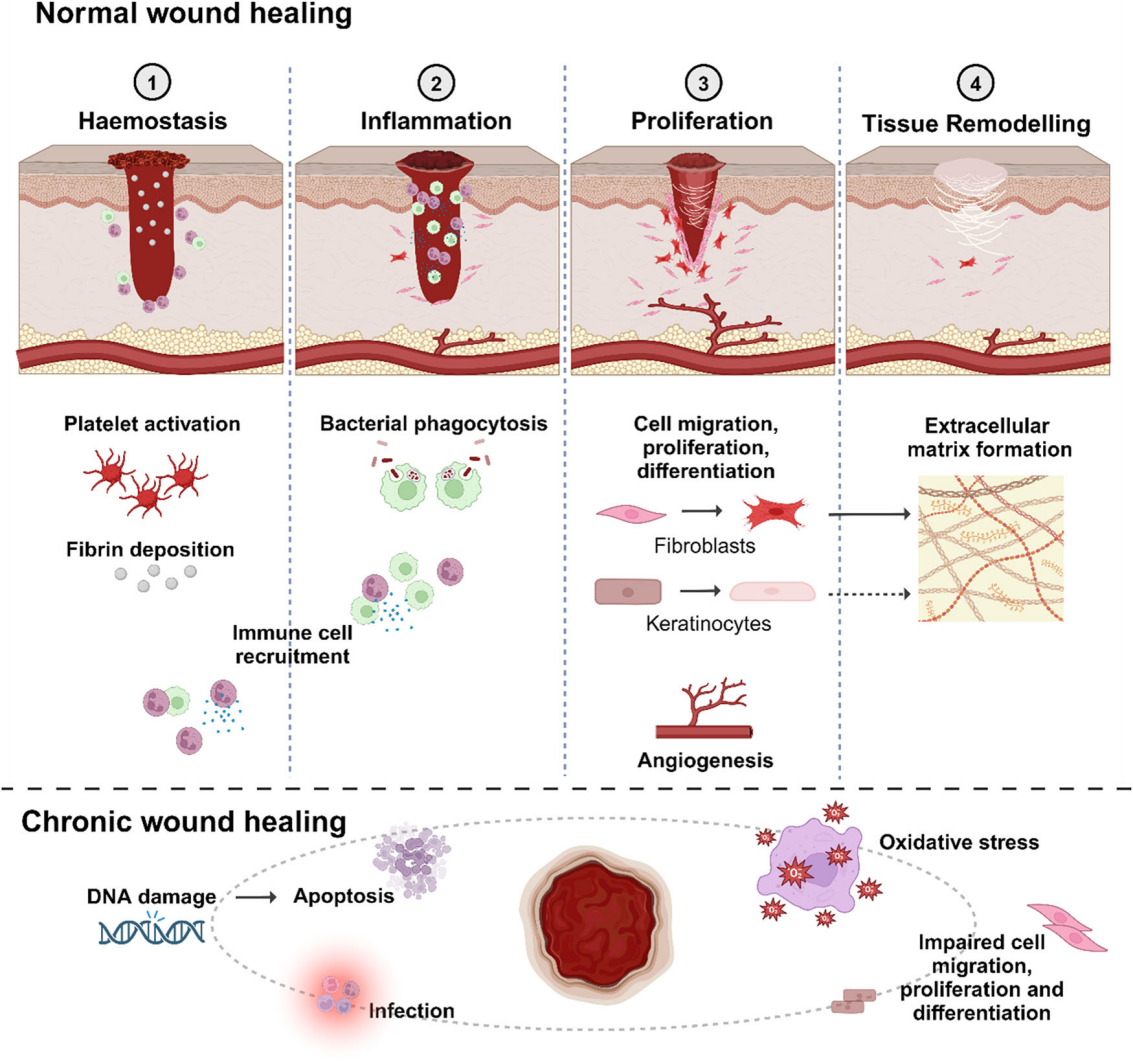
Summary of ROS activities during the wound healing process. Stages of wound healing with illustrations of the various beneficial roles that physiological levels ROS play in the respective stages, in addition to the roles excessive ROS and oxidative stress play in chronic wound pathogenesis. During the haemostasis stage, NO prevents platelet adhesion to vessel walls, whilst ROS such as O_2_^.−^ increases fibrin deposition, and H_2_O_2_ induces the recruitment of monocytes and neutrophils. During inflammation, ROS play important roles in activating immune cells, as well as eliminating pathogens and preventing infection. During the proliferation stage, ROS play vital roles in modulating numerous cellular signalling pathways to promote the proliferation, migration, and differentiation of fibroblasts and keratinocytes, as well as angiogenesis, ultimately promoting collagen remodelling and extracellular matrix formation. Oxidative stress caused by excessive levels of ROS contribute to the pathogenesis of chronic wounds in various ways, including by increasing apoptosis, promoting pathogen expansion and thus infection, as well as impairing the correct modulation of cell signalling pathways involved in cell dynamics.

Reactive oxygen species (ROS) play an essential pleiotropic role in wound healing, and several ROS are involved in the wound healing milieu (Fig. 1). These include superoxide (O_2_^.−^), hydroxyl radicals (.OH) and ions (OH^−^), hydrogen peroxide (H_2_O_2_), and peroxide (.O_2_^−2^)^7^. ROS are implicated in numerous pathophysiological functions within the wound healing process such as anti-bacterial activities^8,9^, as well as acting as secondary messengers in signalling cascades to modulate chemotaxis, angiogenesis, cell growth and migration, stem cell fate, and extracellular matrix (ECM) deposition^7,10–13^.

Importantly, both the levels and timing of ROS production need to be tightly regulated for efficient wound healing^7^. Too high levels caused by either excess ROS production or impaired detoxification lead to oxidative stress, elevated tissue damage, and pathophysiological stalling^14,15^, whilst too low levels impede cellular and molecular processes of wound healing which are dependent on ROS-mediated signal transduction^7^ – ultimately leading to the formation of chronic wounds. Highlighting the delicate and complex balance required, inhibition of ROS has been shown to impair wound healing in numerous animal models^16–23^, whilst improvements in antioxidant capabilities have been shown to be beneficial in treatments of chronic and diabetic wounds^14,24,25^. As such, due to the multifaceted nature of chronic wound pathogenesis and susceptibility to abnormalities in ROS balance, interest in the role of ROS in wound healing, as well as the potential applicability of targeting ROS therapeutically, has grown significantly in recent years^14^. However, it will be essential to further elucidate the precise signalling pathways and mechanisms in which ROS is involved in wound healing. Thus, in this review, we discuss the current knowledge of the cellular and molecular roles of ROS in wound healing and chronic wound pathogenesis, as well as evaluate recent advances in pre-clinical therapeutic approaches targeting ROS and oxidative stress.

## Physiological functions of ROS

**Fig. 2:**
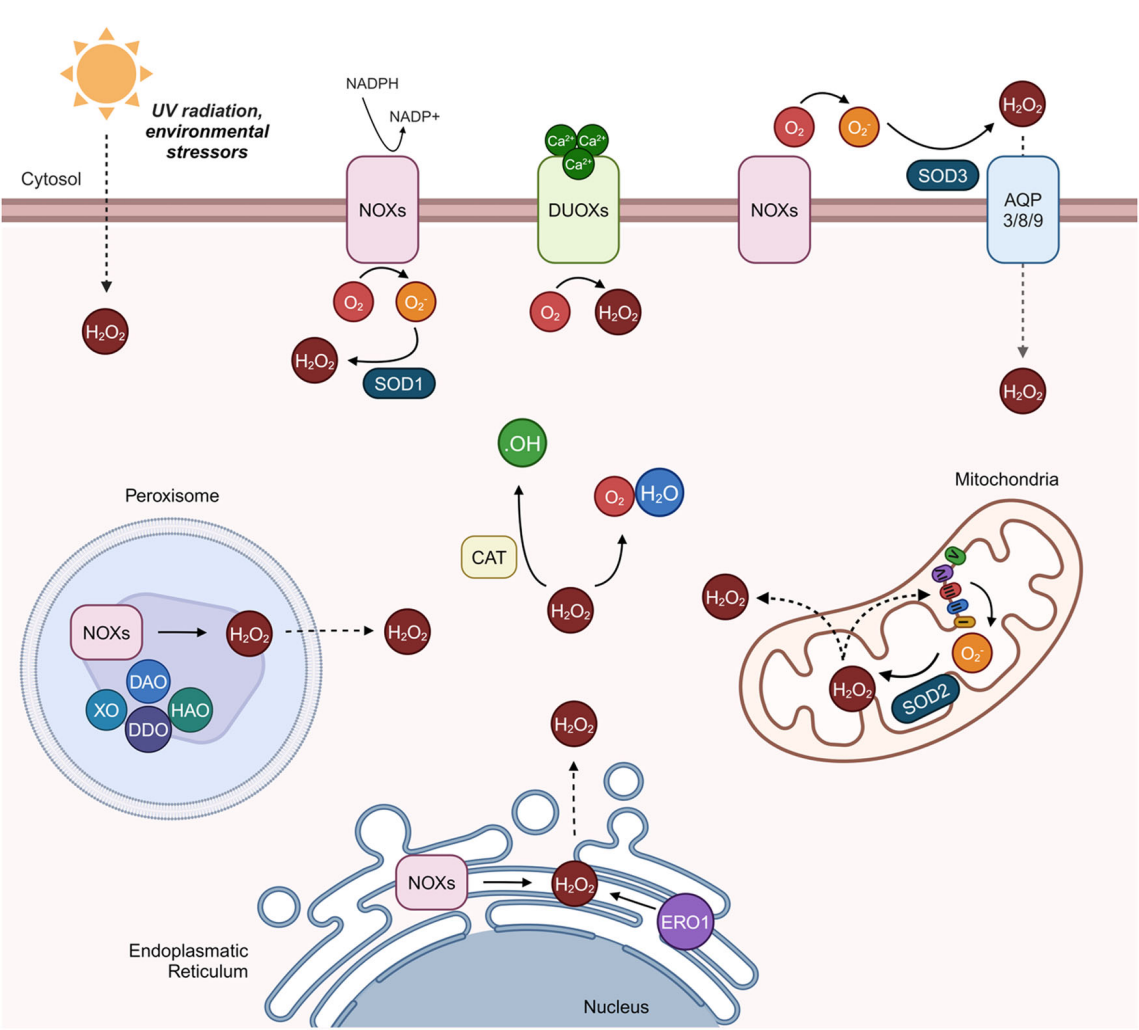
Cellular ROS homeostasis. Schematic diagram depicting the various ROS-generating pathways occurring within cells. At the cell membrane, O_2_^.−^ is converted from O_2_ in an NADPH-mediated reaction by NOXs, which is then converted to H_2_O_2_ by SOD1. H_2_O_2_ can also be produced from O_2_ in a Ca^2+^-mediated reaction by DUOXs or by UV radiation or other environmental stressors. Extracellular H_2_O_2_ can also be imported into cells through AQPs 3, 8, or 9. Within cells, O_2_^.−^ leaks from the ETC during oxidative phosphorylation (OXPHOS) and is converted into H_2_O_2_ by SOD2/MnSOD2 and effluxed out of mitochondria into the cell cytosol. Additionally, H_2_O_2_ can be produced in the ER by either ERO1 or NOXs and effluxed into the cytosol, as can H_2_O_2_ produced by NOXs within peroxisomes. XO, DAO, DDO, and HAO are also produced in peroxisomes. Within the cell cytoplasm, H_2_O_2_ can be detoxified into H_2_O and O_2_ as well as .OH by CAT. ER endoplasmic reticulum, SOD superoxide dismutase, AQP aquaporin, CAT catalase, XO xanthine oxidase, DAO D-amino acid oxidase, HAO 2-hydroxy acid oxidase, ERO ER oxidoreductin.

ROS encompass both free-radical or non-radical derivative (peroxides) oxygen intermediates generated by plasma membrane proteins^26^ (Fig. 2). Physiologically, as well as in pathologies such as wound healing, H_2_O_2_ is recognised as the predominant paracrine ROS secondary messenger involved in signalling cascades^27,28^. This is due to the fact that H_2_O_2_ can quickly and readily diffuse through cell membranes, primarily through aquaporins (AQPs)^29,30^, as well as between neighbouring cells through gap junctions – hemichannels composed of connexins which facilitate the transfer of molecules 1–3 kDa large such as ROS between cells to propagate oxidative signals^31^. Additionally, ROS can directly modulate post-transcriptional gene regulation by interacting and reversibly oxidising thiolate groups and methionine^32^, as well as activating mitosis-related signal transduction pathways^8,33,34^ and electron-rich cysteine residues^35^.

The main sources of intracellular H_2_O_2_ are NADPH oxidases (NOXs) and dual-oxidases (DUOXs)^36–38^, in conjunction with superoxide dismutases (SOD), as well as at the mitochondrial electron transport chain (ETC)^39,40^ – highlighting an important aspect of mitochondria within wound healing^41^. Collectively, NOXs and the ETC generate roughly 85% of H_2_O_2_, with the remaining production deriving from oxidases in the endoplasmic reticulum (ER) and peroxisomes, as well as from cumulative environmental stressors such as UV or ionising radiation^8,40,42,43^. Additionally, membrane-bound NOXs are also responsible for producing .O_2_^.−^ utilised in antimicrobial activities^44^. Other ROS are produced by cytosolic enzymes such as cyclooxygenase^45^, or during lipid metabolism within peroxisomes^46^.

As previously mentioned, whilst low to moderate physiological levels of ROS are beneficial for several processes of wound healing pathophysiology, excess ROS can be deleterious. To counteract these harmful effects, a variety of antioxidant enzymes play vital roles in maintaining ROS levels, termed redox balance. These include peroxiredoxins^47^ such as catalase (CAT)^48^, glutathione peroxidases^49,50^, and mitochondrial nicotinamide nucleotide transhydrogenase (NNT)^51^, which act as ‘sinks’ to remove H_2_O_2_ and maintain non-deleterious physiological levels. In addition, SOD, of which there are four isoforms in humans, are antioxidant metalloproteinases which regulate levels of O_2_^.−^. In particular, the mitochondrial SOD (MnSOD/SOD2) converts .O_2_^.−^ into H_2_O_2_, which is less reactive than .O_2_^.−^ and so can readily be used for cellular signalling. As SOD2 is induced by hypoxia and subsequent HIF-1α activation, it is highly upregulated after wounding^52,53^.

Oxidative stress is the state induced by an overbalance in the form of excess ROS, and can be a causative factor in chronic wound formation^15^, as well as in the pathogenesis of other diseases including cancers, cardiovascular diseases, Parkinson’s, obesity, and other clinically-relevant age-related diseases^54–57^. In the context of wound healing, oxidative stress can lead to the existence of a prolonged pro-inflammatory environment as well as dysregulated re-epithelialisation, as discussed in the following sections^58^.

Finally, another important free radical is nitric oxide (NO), which is a reactive nitrogen species (RNS) involved in vascularisation, inflammation, and antimicrobial activities in wound healing^59–61^. Most importantly with regards to wound healing, NO plays an important role in pathogen clearance during the inflammatory stage, and this NO is produced by the inducible nitric oxide synthase (iNOS) isozyme^62,63^. Here, NO targets both gram-negative and -positive bacteria through aberrant peroxidation and the production of ONOO^-^, although this can be hindered by its short half-life^64,65^. Alternatively, lower levels of NO, produced by endothelial nitric oxide synthase (eNOS), play important roles in preventing platelet adhesion to vessel walls during haemostasis^66^, and for both keratinocyte and fibroblast proliferation, motility, and differentiation at the later proliferation and tissue remodelling stages of wound healing^67–69^. Insufficient production of NO has been shown to be a significant factor in the development of chronic wounds such as diabetic foot ulcer (DFU), primarily due to resultant impaired antimicrobial activities^63,70^.

## ROS and immune cell function during wound healing

### ROS and leucocyte recruitment

Immediately following skin wounding there is a peak in ROS production to ~0.5–50 μM. Here, ROS are utilised to simultaneously recruit leucocytes to the wound site, as well as to induce vasoconstriction^16,17,71^. Through studies of ROS dynamics in embryonic zebrafish wounding, Niethammer et al. demonstrated for the first time that epithelial cell production of H_2_O_2_ preceded the recruitment of leucocytes, and in particular that DUOX was the main source of H_2_O_2_ at the wound site and inducer of rapid leucocyte recruitment from long distances^17^. In embryonic *Drosophila*, the activation of DUOX and subsequent H_2_O_2_ production was shown to be triggered by wound-induced calcium (Ca^2+^) flashes, where Ca^2+^ binds to an EF hand Ca^2+^-binding motif of DUOX^72^.

Expanding on this work, Yoo et al. demonstrated the cystine residue C466 on the Src family kinase Lyn as being the direct target of H_2_O_2_ to induce neutrophil recruitment to the wound site, mediated through ERK signalling^16^. Alternatively, the activation of DUOXs, with subsequent H_2_O_2_ production and neutrophil recruitment, can also be activated by ATP through the P2Y receptor (P2YR)/phospholipase C (PLC) Ca^2+^ signalling pathway following wounding in embryonic zebrafish tailfins^73^.

### ROS and macrophage function

Macrophages play important roles within the wound healing process, including in antimicrobial activities, as well as inflammation, angiogenesis, anti-inflammation, re-epithelialisation, and tissue resolution^74^. ROS-induced HIF-1α stabilisation leads to the activation of macrophages in the early stages of wound healing, and promotes metabolic reprogramming towards glycolysis^75,76^, as well as increased angiogenesis^76^. Importantly, both NOX1- and NOX2-produced ROS are required for the activation and differentiation of monocytes into proinflammatory M1 and anti-inflammatory M2 macrophages^77^. Additionally, in atherosclerotic lesions, NOX4-produced ROS drives monocyte and macrophage cell death^77^ – a vital step required to prevent prolonged inflammation in wound healing^78^.

Although NOXs are essential for macrophage activation, they have been shown to be dispensable for M1 macrophage-mediated proinflammatory cytokine production^79^. Instead, pro-inflammatory cytokine production and inflammasome activation in macrophages predominantly relies on the regulation of the Nrf2 response^80^, which can be primarily activated by glutathione or thioredoxin systems, as well as to a lesser extent NOXs^81^. Other important pro-inflammatory signalling pathways regulated by ROS – and in particular H_2_O_2_ – include p38-MAPK-mediated NF-κB/HIF-1α^82,83^, and JAK-STAT pathways^84^.

Monoamine oxidases (MAOs) – a mitochondrially-located enzyme responsible for catalysing the oxidative deamination of H_2_O_2_^85^ – is upregulated by the M2 macrophage-activating IL-13 and IL-4, or LPS signals. This process is mediated through JAK signalling pathways and is thus important in anti-inflammation and re-epithelialisation during wound healing^86^. Indeed, MAO inhibitors significantly reduced H_2_O_2_ levels and NF-κB/TNFα activation to impair apical migration and proliferation of junction epithelium in a rat chronic wound model^87^. Alternatively, DUOX-induced ROS stimulated the activation of macrophages and promoted epithelial proliferation in a JNK-dependent manner during *Drosophila* epithelial disc healing^88^.

Of note, whilst many studies have demonstrated the importance of ROS and macrophage function in other pathologies and physiological contexts, few have specifically investigated this link within wound healing pathophysiology^89^. Importantly, the majority of studies in this area used the oversimplistic M1 and M2 macrophage classifications, whereas in recent years advancements have been made to study the more elaborate classifications of macrophages in the general immunology setting^90^, and this should thus be applied to the macrophages in wound healing setting^91^. Single-cell RNA-sequencing (scRNAseq) and other advanced omics-based techniques have in recent years significantly powered the investigation of the roles of specific cell lineages, including macrophage lineages, in different stages of wound healing^76,92^. As such, future investigations seeking to elaborate on the roles of ROS dynamics in wound healing would greatly benefit from the utilisation of these techniques.

### ROS and antimicrobial activities

**Fig. 3:**
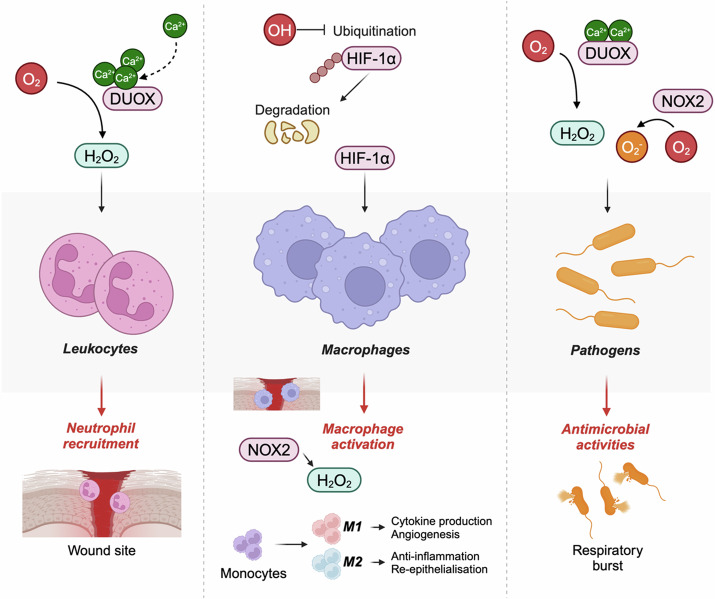
Roles of ROS utilisation in immune cells during wound healing. Schematic diagram depicting the roles of ROS in neutrophil and macrophage recruitment, as well as in antimicrobial activities during the inflammatory stage of wound healing. Here, wounding-induced Ca^2+^ flashes lead to the upregulation of DUOX-mediated H_2_O_2_ production, stimulating the recruitment of leukocytes to the wound site. Additionally, OH prevents the ubiquitination and subsequent degradation of HIF-1α, leading to increased HIF-1α signalling and macrophage activation, primarily mediated through H_2_O_2_ signalling. Finally, immune cells utilise various reactive oxygen species, including O_2_^−^, to destroy pathogens through respiratory bursts.

One of the major factors that leads to the formation of chronic wounds is that of prolonged inflammation and pathogenic infection, which can hamper angiogenesis, stem cell function, and extracellular matrix remodelling^93^. During acute wound healing, immune cells are required to eliminate pathogens and prevent infection at the wound site, and ROS play an essential role in this function^94^ (Fig. 3). Here, extracellular H_2_O_2_ – generated by DUOXs in response to the increased Ca^2+^ binding following wounding – promotes the production of bacteria-destroying ROS after reacting with halide or thiocyanate^95,96^. In the absence of this function, polymicrobial biofilms form, which can lead to the expansion of a pathogenic environment and ultimately the stalling of the wound healing response and thus chronic wound formation^97^.

In a dual action, peroxidases such as myeloperoxidase and eosinophil peroxidase convert H_2_O_2_ into other oxidants such as hypochlorous acid, which is then used by neutrophils in antibacterial activities^98,99^. This process additionally prevents the toxic build-up of H_2_O_2_ which can occur in the early stage of wound healing following leucocyte recruitment, where H_2_O_2_ levels are highest^100,101^.

Macrophages also play an important role in clearing pathogens during wound healing through phagocytosis. Here, NOX2-derived O_2_^.-^ is released into phagocytotic vesicles to kill internalised pathogens through respiratory bursts^89^.

## Role of ROS in re-epithelialisation

### ROS in platelet aggregation and angiogenesis

Moderate amounts of ROS (up to a 40% increase) are required for the reduction in platelet adhesion to collagen surfaces and thus platelet activation^102,103^. In light of this, and conversely, whilst ROS is known to accelerate platelet function in wound healing, transfer of platelet-derived mitochondria into diabetic mice improved wound healing in part by preventing the overexpression of ROS^104^. Importantly, H_2_O_2_ induces the recruitment of vascular smooth muscle cells to the wound site^11,105^.

**Fig. 4:**
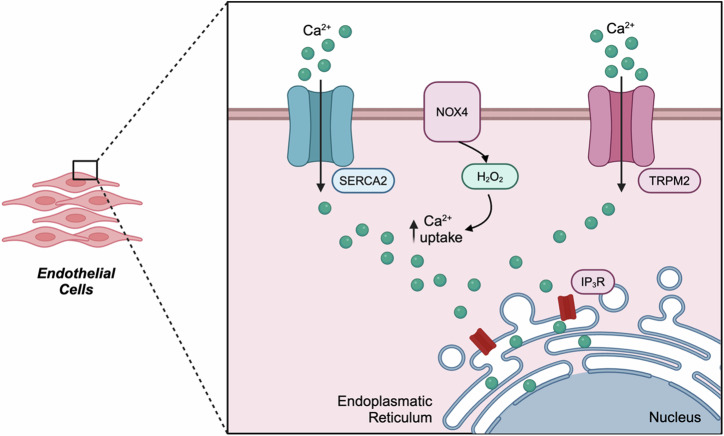
ROS and endothelial cell function. Schematic depicting how H_2_O_2_ produced by NOX4 increases Ca^2+^ uptake into endothelial cells through elevated SERCA2 and TRPM2 channel activity, subsequently leading to increased endothelial cell division and migration – thereby promoting angiogenesis.

As previously mentioned, NO plays an important role in angiogenesis during wound healing. Here, elevated NO production – as a result of increased activation of NOXs, in particular NOX4 – leads to the stabilisation of HIF-1α and thus promotion of endothelial cell (EC) survival, migration, differentiation, and therefore neovascularisation^64,106–108^. Highlighting this, near-infrared (NIR)-triggered NO production supressed the proteasomal degradation of HIF-1α. Here, by preventing the interaction of HIF1-α with E3 ubiquitin ligases, both VEGF and CD31 expression was enhanced in ECs, coinciding with increased cell proliferation and migration – collectively accelerating wound healing in diabetic mice^64^. H_2_O_2_ produced by NOX4 also activated both the TRPM2^109^, and SERCA2 channels^110^ to promote Ca^2+^ uptake and thus improve EC activity (Fig. 4). In wound healing and other hypoxic-state pathologies such as brain ischemia, these ROS-derived effects on ECs are associated with phosphorylation-dependent activation of various signalling molecules including those of ERK, c-JUN, MAPK, AKT, SMAD, and JNK^111^.

Finally, production of O_2_·^−^ by both NOX2 and NOX4 also leads to the upregulation of VEGF^110,112^. In particular, NOX2 was demonstrated to stimulate VEGFR2 and angiogenesis in wounds through the activation of NF-κB by 2-deoxy-D-ribofuranose 1-phosphate (dRP) – an intermediate of pyrimidine metabolism. This NOX2-derived ROS was primarily generated by both platelets and macrophages^13,113^.

### H_2_O_2_ mediated cell signalling and re-epithelialisation

Many cytoskeletal proteins possess cysteines which are highly sensitive to oxidation^114^, and in a complementary fashion, production of H_2_O_2_ occurs primarily in leading edge cells involved in re-epithelialisation^115^. In particular, H_2_O_2_ promotes actin cytoskeleton reorganisation and cell migration by directly oxidising actin and actin-binding proteins^116^, as well as activating numerous cell signalling pathways associated with re-epithelialisation.

**Fig. 5:**
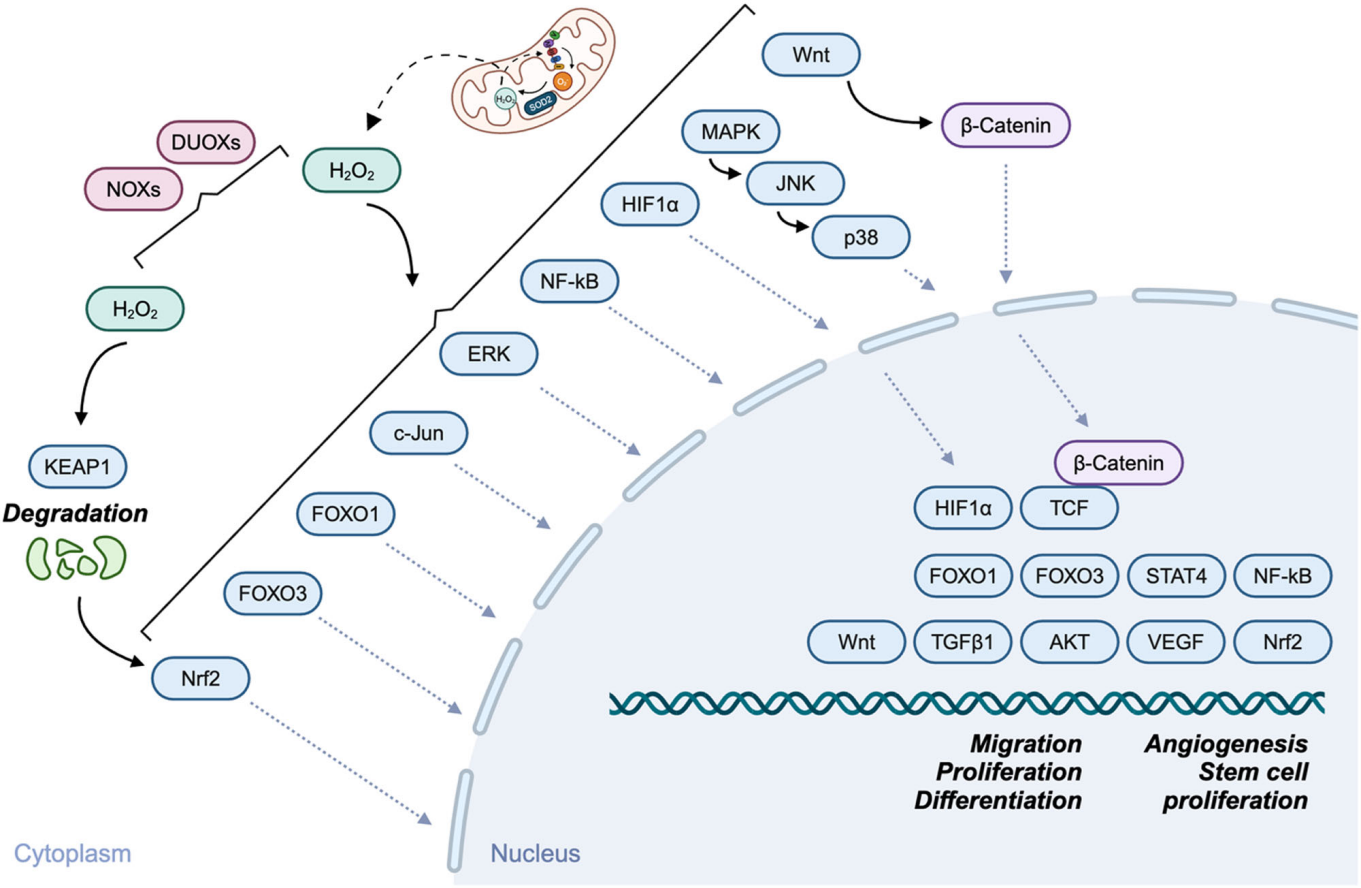
ROS and re-epithelialisation. Schematic showing the various signalling molecules and pathways which are modulated by ROS to regulate re-epithelialisation during wound healing. H_2_O_2_ produced by either NOXs or DUOXs, or derived from mitochondria, stimulate numerous cell signalling pathways which ultimately lead to the upregulation of processes to accelerate wound healing – such as cell migration, proliferation, differentiation, angiogenesis, or stem cell propagation.

As discussed previously, there are several mechanisms in which ROS-mediated signalling cascades are initiated in wound healing (Fig. 5). Indeed, ROS production required for both immune cell function and re-epithelialisation share similar stimuli. In one key example, Hunter et al. demonstrated in *Drosophila* embryo healing that mitochondrially-derived H_2_O_2_ – produced downstream of intracellular Ca^2+^ bursts – led to the polarisation of the actomyosin cytoskeleton and E-cadherin distribution around the wound to promote wound healing^117^. Specifically, this action occurred via oxidation of the Src kinase Src42, and supported results from a previous study in which mitochondrial ROS (mtROS) was produced downstream of Ca^2+^ bursts following wounding^118^. In addition to Ca^2+^-mediated activation, DUOX can also be activated downstream of extracellular ATP-activated purinergic receptors^119–121^.

One cell signalling pathway regulated by wounding-induced ROS is that of c-JUN. Here, the inhibition of wounding-induced ROS accumulation significantly inhibited healing in planarian worms by preventing F-actin reorganisation and epithelial cell rearrangements – mediated through c-JUN activation at the wound site^122^. Separately, ROS-mediated activation of c-JUN also accelerated wound healing in diabetic rats through increases in angiogenesis and re-epithelialisation^123^, whilst NOX-produced H_2_O_2_-activation of the JNK pathway increased epithelial cell proliferation in adult zebrafish tailfin healing^19^.

Alternatively, H_2_O_2_ also regulates MAPK signalling during wound healing – namely, through thioredoxin (Trx) oxidation and PI3K/AKT1-Ask1-MAP3K-mediated activation of JNK and p38^22,26,124^. Interestingly, in a study investigating the interplay between ROS and AKT signalling in *Drosophila* regeneration, the importance of nutrient sensing and metabolism in this pathway was highlighted^125^. Here, ROS-mediated phosphorylation of Ask1 at Ser38 induced p38-mediated regeneration in a nutrient-sensitive insulin signalling manner, supporting similar findings in studies of stress-induced regeneration in the gut^126^. As metabolic regulation of fibroblasts and keratinocytes during re-epithelialisation is known to be important^127^, future studies should aim to further investigate the role of the MAPK and other relevant signalling pathways.

Another pathway that H_2_O_2_ has been shown to be important during the proliferation stage of zebrafish regeneration is that of hedgehog signalling^19,128,129^. A recent paper using the zebrafish tailfin regeneration model demonstrated that Sonic hedgehog (Shh) – a key signalling protein in the hedgehog signalling cascade – acted downstream of NOX to increase SOD activity and H_2_O_2_ production in the early stage of tailfin regeneration, most likely due to SOD oxidation^130^. Interestingly, although not fully elaborated on, the authors also demonstrated binding sites for HIF1α, STAT3, and NF-κB in *Shha*, suggesting that this kinase may additionally act on these factors to regulate redox balance during wound healing^130^. Importantly, Hedgehog signalling acts in close synergy to canonical Wnt signalling in regeneration as well as other pathological contexts^129,131^. In a separate study, NOX-induced H_2_O_2_ activated Wnt/β-catenin signalling and induced *FGF20* transcriptional activation to promote epidermal regeneration^18^. Canonical Wnt signalling and mitochondrial H_2_O_2_ production was also downregulated in a mouse model with *TFAM* KO, with subsequent defects in epidermal differentiation due to the inhibition of Notch signalling^132^.

As well as its previously mentioned functions during the inflammation stage of wound healing, NF-κB has been shown to play significant roles during re-epithelialisation. Of note, early ROS signalling following embryonic zebrafish tailfin amputation led to the activation of NF-κB as well as promoter activation of vimentin. Here, vimentin promoted collagen formation and organisation^133^. Through serine/tyrosine phosphorylation and ubiquitination, H_2_O_2_ acts as a regulator of IκB kinases by inducing their proteolytic degradation^134,135^, which subsequently activates NF-κB and therefore promotes angiogenesis and re-epithelialisation. NF-κB is also directly regulated by H_2_O_2_-oxidation of cysteine residues in its DNA-binding region^136^. ROS has also been shown to increase the expression of TGF-β1 signalling, with downstream effects including elevated expression of collagen, fibronectin, bFGF, and matrix production^104,137^. As mitochondria are important in both TGF-β1 signalling and collagen organisation^138^, as well as that of general ROS regulation during wound healing, future studies should aim to explore the potential link between these factors. Indeed, the link between ROS and TGF-β1-induced epithelial-mesenchymal transition (EMT) has been demonstrated in endometrial cell pathology^139^.

H_2_O_2_ was shown to accelerate re-epithelialisation in embryonic zebrafish tailfins following wounding via the Src family kinase (SFK) FYN^140^. Although not confirmed in the particular study, FYN is known to be involved in keratinocyte differentiation^141,142^. Of note, this study confirmed findings from other studies whereby wounding led to the activation of Ca^2+^, ERK, and H_2_O_2_-mediated signalling cascades independently of each other^140^.

FOXO are transcription factors important in wound healing, and can either be activated by direct cysteine oxidisation, or in response to upstream redox signalling^143^. In particular, FOXO1 regulates cell cycle progression, apoptosis, and angiogenesis^144,145^, and promoted both in vivo and in vitro wound healing by preventing oxidative stress in keratinocytes via the upregulation of GPX-2 and GADD45α – thus maintaining TGF-β1 mediated migration and apoptosis inhibition^146^. Additionally, low level (10 µM) H_2_O_2_ treatment induced JNK-mediated FOXO3a translocation and activation to promote AKT-mediated stem cell proliferation, which when transplanted, increased re-epithelialisation and angiogenesis in wounded mice^147^. Here, H_2_O_2_ promoted the production of CAT, SOD2, GPX1, and GPX2, collectively increasing stem cell proliferation and preventing oxidative stress.

Nrf2 is a cytoprotective transcription factor which is upregulated in wound healing and acts to restore redox balance by increasing levels of various antioxidants^148,149^. In physiological conditions, Nrf2 is ubiquitinated and therefore inactivated by KEAP1. However, oxidation of cysteine residues on KEAP1 – including Cys151, Cys273, and Cys288 – by H_2_O_2_ induces a conformational change in KEAP1 and stimulates the activation of Nrf2^150^. Although not studied specifically at the molecular level, the antioxidant activities of Nrf2 play important roles in wound healing – regulating apoptosis, metabolism, autophagy, angiogenesis, as well as cell proliferation and migration^151^. Various studies have attempted to utilise Nrf2-modulating compounds for chronic wound treatment^152–157^. However, more research into the specific molecular mechanisms is required in order to further elucidate the specific cellular and molecular role of Nrf2 in wound healing pathophysiology.

Finally, DUOX1-mediated H_2_O_2_ production was also shown to be important for peripheral sensory axon reinnervation in zebrafish tailfin wound healing – highlighting another import role that ROS play, even in more uncommonly studied aspects of wound healing^158^.

Interestingly, by comparing ROS expression in both wound healing (early stage, lower levels) and regeneration (later stage, elevated levels) in planarians, Van Huizen et al. demonstrated that different levels of ROS act upstream of signalling pathways in a threshold-dependent manner to dictate the type of response required^122^. Similar results were found when investigating zebrafish, whereby simple wound healing required only early accumulation of ROS for JNK pathway activation, whilst injuries which necessitated new tissue formation required sustained H_2_O_2_ production, activating apoptosis pathways as well as JNK^19,130^.

### Chronic wounds – redox balance abnormalities and current advancements in treatment strategies

A balanced state of oxidative stress is essential for normal wound healing. While physiological levels of ROS are required in the normal transition between wound healing phases, as previously described, an overproduction of ROS has deleterious effects and can hinder wound healing^159^. Multiple molecular mechanisms can explain this effect. For example, healing can be hindered by increased tissue damage, via opposing effects of cytokines such as VEGF and TNFα^160,161^. Excessive ROS can also alter and degrade extracellular matrix proteins and impair the function of keratinocytes and fibroblasts^162^.

Diabetic wounds, another category of hard-to-heal wounds, are complex and multifactorial. Their aetiology consists classically of a triad of neuropathy, impaired vascularisation and higher susceptibility to infection. These wounds are also notoriously affected by tissue injury after prolonged hypoxia and excessive oxidative stress^93^. It is thus not surprising that targeting ROS has emerged as a potential therapy for hard-to-heal wounds, similar to other diseases, such as cancer^163,164^, neurodegenerative diseases^165^, T cell-mediated autoimmune diseases^166^, inflammatory skin diseases^167^, and others. Specifically regarding cancer, ROS play similar pleiotropic roles as they do in wound healing and chronic wound pathogenesis. Depending on the type of cancer and stage, this can include hypoxia-related ROS functions and signalling pathways such as PI3K/AKT or MAPK/ERK pathways, among others, to promote proliferation, migration, or angiogenesis. Similarly, tight regulation of ROS levels is required for cancer progression and can thus also be potentially targeted therapeutically^164^.

Another factor to take into account is that of senescence, which is the phenomenon of cell cycle arrest and the inhibition of cell proliferation, and is a hallmark of several age-related pathologies, including in the pathogenesis of some chronic wounds^168^. ROS accumulation and oxidative stress can accelerate senescence in both fibroblasts^169^ and endothelial cells^170^, whilst UV-induced ROS upregulation additionally increases senescence and photoageing in skin^171^.

Diverse strategies have emerged to modulate ROS in wound healing, namely the use of antioxidant materials such as *N*-acetyl-l-cysteine (NAC)^172^ or enzymes which either increase local perfusion such as glucose oxidase, or clear free radicals through SODs^173,174^. Biocompounds that target ROS have also been implicated in improved wound healing, mediated by their effects on perfusion, cell migration, and ROS suppression. Examples of these molecules are Resolvin E1, PDGF, Galectin-1, Alpha-arbutin and Nicotinamide^175–179^. Nanoparticles have also been developed to improve wound healing, mainly via ROS scavenging, anti-inflammatory and antibacterial effects, and applied to several in vitro and in vivo models^180–184^, as well as other molecules^185–188^.

On the other hand, increased angiogenesis has been demonstrated when wounds were treated topically with H_2_O_2_^189^ and both hyperbaric and topical oxygen led to accelerated wound healing^190–192^. The array of both pro- and anti-ROS treatment stratagies that have been applied to the study of wound healing are described in detail in Table 1.

**Table 1:** Molecules targeting ROS in wound healing

Category	Material	Effect	Model	Ref
Antioxidants	NAC	Reduces ROS levels and improves cell migration and proliferation	Cultured human gingival fibroblasts in a high-glucose environment	^172^
Enzymes	Glucose oxidase	Increases perfusion (via NO); collagen formation	Diabetic mice with full-thickness wounds, applied in wound dressings	^173^
	Superoxide dismutase	Clearance of free radicals	Hydrogels used in diabetic rat models with full-thickness wounds	^174^
Bio compounds	Resolvin E1	Promotes intestinal wound repair (via CREB, mTOR, Src-FAK)	Murine biopsy-induced colonic mucosal wounds	^179^
	PDGF	Increases NO (higher perfusion); increased angiogenesis and cell migration	Rat model with excisional wounds, mice lacking PDGF receptors/ligands	^175,176^
	Galectin-1	Effect on myofibroblast function and signalling with the release of ROS (via NOX)	Mice injected with recombinant Galectin-1 protein	^177^
	Alpha-arbutin	Promotes healing via upregulation of IFG1R	Cultured human dermal fibroblast	^178^
	Nicotinamide	Suppresses ROS; increases cell motility	Cultured human HaCaT keratinocytes	^198^
ROS intermediates	Topical H_2_O_2_	Converts into available O_2_, increase angiogenesis	Guinea pigs with ischemic wounds	^189^
Oxygen	Hyperbaric O_2_	Reduces wound hypoxia; increases fibroblast proliferation, angiogenesis and accelerated wound healing	Diabetic mouse model, in vitro model, patients (with diabetic and miscellaneous wounds)	^190–192^
	Topical O_2_	Reduces wound hypoxia; accelerates wound healing	Patients, meta-analyses	^199^
Nanomaterials	Copper-based nanoenzymes	ROS scavenging at low concentrations; promotes re-epithelialization and granulation	Murine diabetic model with full-thickness wounds	^183^
	Cerium oxide nanoparticles	ROS scavenging; stimulation of proliferation and migration of endothelial cells, keratinocytes, and fibroblasts	Human keratinocytes and microvascular endothelial cells. Mouse fibroblasts. Full-thickness wounds in mouse model	^181^
	PDA	Capacity of scavenging free radicals; energy transfer	PDA hydrogels in dental pulp stem cells, rat model	^182^
	Gold nanoparticles	Anti-inflammatory; antioxidation; enhanced wound healing	In combination with antioxidative small molecules, diabetic mouse model	^180^
	Selenium nanoparticles	Antibacterial; anti-inflammatory; antioxidation	In combination with hydrogels, mouse model with full-thickness wounds	^184^
Others	Carbon quantum dots	Eliminates ROS; reduces oxidative stress	Carbon dot hydrogel applied in infected wound from a mouse model	^188^
	Galvanic particles	Enhance production of ROS by keratinocytes; reduced inflammation; increased fibroblast migration	Human keratinocytes, dermal fibroblasts	^185,186^
	Prussian blue	Reduces ROS; increased collagen deposition; induces keratinocyte differentiation, neovascularization, and wound closure	Topically applied, mouse model	^187^

Summary of ROS modulating materials, their effect or known mechanism of action, and the models where they were tested.

*NAC* *N*-acetyl-l-cysteine, *PDGF* platelet-derived growth factor, *IFG1R* insulin-like growth factor 1 receptor, *PDA* polydopamine.

There is, however, a lack of understanding of the exact underlying mechanisms behind the effect of these molecules in many studies and a lack of validation in human samples, which may explain the scarcity of clinical translation. It is also not always evident in the course of a chronic wound history when there is a need to reduce or stimulate ROS production, hence the importance of allying possible treatment strategies that target ROS with the use of sensors that could translate the actual needs of the wound at that given moment^193^. The utilisation of omics approaches such as scRNAseq to delineate the specific effects of ROS modulation in individual cell types during different stages of wound healing and chronic wound pathogenesis would additionally be of benefit for this purpose.

## Conclusions and perspective

ROS and their essential roles in cellular signalling regulation is one of the most important components of the complex pathophysiology of the wound healing cascade. However, owing to the multifaceted nature of wound healing and chronic wound formation, improper regulation of either ROS production or removal can lead to oxidative stress and impairment of wound healing, contributing to the formation and propagation of chronic wounds. In light of this, there has been a greater emphasis in recent years towards investigating whether ROS can be targeted to either accelerate wound healing, or conversely, be used as a treatment for chronic wounds – with no concrete advancements with regards to clinically approved therapies as of yet.

This discrepancy can partially be explained by gaps in knowledge surrounding the cellular and molecular landscape of ROS in wound healing, including for example, the role of ROS and metabolism^76,127^, as well as that of redox signalling and stem cell homeostasis. In addition, future work is required in order to elucidate the specific dynamics of ROS and in particular H_2_O_2_, including the movement of H_2_O_2_ and its interplay with specific AQPs and gap junctions, or the role of ROS and other ROS-producing organelles such as the ER, which play important roles in wound healing and may potentially be targeted through pre-clinical agents^194^.

Finally, it is important to note that whilst most of the investigations into the role of ROS in wound healing have been performed in animal models such as zebrafish or *Drosophila*, these studies have predominantly investigated the similar but pathophysiologically different process of regeneration, as opposed to wound healing itself^195^. However, the fact that various studies have shown that many of these pathways are activated in separate animal models^140,196,197^ – such as ERK signalling – suggests that the effects of ROS are phylogenetically conserved. However, advances in methods which allow for more targeted analyses of specific cell types and their roles in different stage of wound healing pathophysiology, such as scRNAseq, are an important step in advancing knowledge in the field of ROS and wound healing.

## Supplementary information

Transparent Peer Review file

## References

[1] Peña, O. & Martin, P. Cellular and molecular mechanisms of skin wound healing. *Nat. Rev. Mol. Cell Biol*. **25**, 599–616 (2024). 10.1038/s41580-024-00715-1 38528155

[2] Torres, M. et al. The temporal dynamics of proteins in aged skin wound healing and comparison to gene expression. *J. Invest. Dermatol.* (2024). 10.1016/j.jid.2024.09.024 39675661

[3] Schultz, G. et al. In *Principles of Wound Healing*. (University of Adelaide Press, 2011). 30485016

[4] Eming, S., Krieg, T. & Davidson, J. Inflammation in wound repair: molecular and cellular mechanisms. *J. Invest. Dermatol.* **127**, 514–525 (2007). 17299434 10.1038/sj.jid.5700701

[5] Nirenjen, S. et al. Exploring the contribution of pro-inflammatory cytokines to impaired wound healing in diabetes. *Front. Immunol.* **14**, 1216321 (2023). 37575261 10.3389/fimmu.2023.1216321 PMC10414543

[6] Evans, J., Kaitu’u-Lino, T. & Salamonsen, L. Extracellular matrix dynamics in scar-free endometrial repair: perspectives from mouse in vivo and human in vitro studies. *Biol. Reprod.* **85**, 511–523 (2011). 21613633 10.1095/biolreprod.111.090993

[7] Dunnill, C. et al. Reactive oxygen species (ROS) and wound healing: The functional role of ROS and emerging ROS-modulating technologies for augmentation of the healing process. *Int. Wound J.* **14**, 89–96 (2017). 26688157 10.1111/iwj.12557 PMC7950185

[8] Sies, H. & Jones, D. Reactive oxygen species (ROS) as pleiotropic physiological signalling agents. *Nat. Rev. Mol. Cell Biol.* **21**, 363–383 (2020). 32231263 10.1038/s41580-020-0230-3

[9] Polaka, S., Katare, P. & Pawar, B. & et, a. Emerging ROS-modulating technologies for augmentation of the wound healing process. *ACS Omega* **7**, 30657–30672 (2022). 36092613 10.1021/acsomega.2c02675 PMC9453976

[10] Schieber, M. & Chandel, N. ROS function in redox signaling and oxidative stress. *Curr. Biol.* **24**, R453–R462 (2014). 24845678 10.1016/j.cub.2014.03.034 PMC4055301

[11] Klyubin, I. et al. Hydrogen peroxide-induced chemotaxis of mouse peritoneal neutrophils. *Eur. J. Cell Biol.* **70**, 347–351 (1996). 8864663

[12] Li, X. et al. Exosomes from adipose-derived stem cells overexpressing Nrf2 accelerate cutaneous wound healing by promoting vascularization in a diabetic foot ulcer rat model. *Exp. Mol. Med.* **50**, 1–14 (2018). 29651102 10.1038/s12276-018-0058-5 PMC5938041

[13] Vara, D. et al. Direct Activation of NADPH Oxidase 2 by 2-Deoxyribose-1-Phosphate Triggers Nuclear Factor Kappa B-Dependent Angiogenesis. *Antioxid. Redox Signal* **28**, 110–130 (2018). 28793782 10.1089/ars.2016.6869 PMC5725637

[14] Wang, G. et al. The initiation of oxidative stress and therapeutic strategies in wound healing. *Biomed. Pharmacother.* **157**, 114004 (2023). 10.1016/j.biopha.2022.114004 36375308

[15] Cano Sanchez, M., Lancel, S., Boulanger, E. & Neviere, R. Targeting Oxidative Stress and Mitochondrial Dysfunction in the Treatment of Impaired Wound Healing: A Systematic Review. *Antioxidants* **7**, 98 (2018). 10.3390/antiox7080098 PMC6115926 30042332

[16] Yoo, S., Starnes, T., Deng, Q. & Huttenlocher, A. Lyn is a redox sensor that mediates leukocyte wound attraction in vivo. *Nature* **480**, 109–112 (2011). 22101434 10.1038/nature10632 PMC3228893

[17] Niethammer, P., Grabher, C., Look, A. & Mitchison, T. A tissue-scale gradient of hydrogen peroxide mediates rapid wound detection in zebrafish. *Nature* **459**, 996–999 (2009). 19494811 10.1038/nature08119 PMC2803098

[18] Love, N. et al. Amputation-induced reactive oxygen species are required for successful Xenopus tadpole tail regeneration. *Nat. Cell Biol.* **15**, 222–228 (2013). 10.1038/ncb2659 PMC3728553 23314862

[19] Gauron, C. et al. Sustained production of ROS triggers compensatory proliferation and is required for regeneration to proceed. *Sci. Rep.* **3**, 2084 (2013). 10.1038/srep02084 PMC3694286 23803955

[20] Al Haj Baddar, N., Chithrala, A. & Voss, S. Amputation-induced reactive oxygen species signaling is required for axolotl tail regeneration. *Dev. Dyn.* **248**, 189–196 (2019). 30569660 10.1002/dvdy.5 PMC6340771

[21] Labit, E. et al. Opioids prevent regeneration in adult mammals through inhibition of ROS production. *Sci. Rep.* **8**, 12170 (2018). 10.1038/s41598-018-29594-1 PMC6093857 30111876

[22] Santabárbara-Ruiz, P. et al. Ask1 and Akt act synergistically to promote ROS-dependent regeneration in Drosophila. *PLoS Genet* **15**, e1007926 (2019). 30677014 10.1371/journal.pgen.1007926 PMC6363233

[23] Gouzos, M. et al. Antibiotics Affect ROS Production and Fibroblast Migration in an I*n-vitro* Model of Sinonasal Wound Healing. *Front. Cell Infect. Microbiol* **10**, 110 (2020). 32266162 10.3389/fcimb.2020.00110 PMC7096545

[24] Xu, Z., Han, S., Gu, Z. & Wu, J. Advances and Impact of Antioxidant Hydrogel in Chronic Wound Healing. *Adv. Healthc. Mater.* **9**, e1901502 (2020). 10.1002/adhm.201901502 31977162

[25] Dong, Y. & Wang, Z. ROS-scavenging materials for skin wound healing: advancements and applications. *Front. Bioeng. Biotechnol*. **11**, 1304835 (2023). 10.3389/fbioe.2023.1304835 PMC10749972 38149175

[26] Checa, J. & Aran, J. Reactive Oxygen Species: Drivers of Physiological and Pathological Processes. *J. Inflamm. Res.* **13**, 1057–1073 (2020). 33293849 10.2147/JIR.S275595 PMC7719303

[27] Di Marzo, N., Chisci, E. & Giovannoni, R. The Role of Hydrogen Peroxide in Redox-Dependent Signaling: Homeostatic and Pathological Responses in Mammalian Cells. *Cells* **7**, 156 (2018). 30287799 10.3390/cells7100156 PMC6211135

[28] Santos, C., Hafstad, A. & Beretta, M. & al., e. Targeted redox inhibition of protein phosphatase 1 by Nox4 regulates eIF2α-mediated stress signaling. *EMBO J.* **35**, 319–334 (2016). 26742780 10.15252/embj.201592394 PMC4741303

[29] Bienert, G., Schjoerring, J. & Jahn, T. Membrane transport of hydrogen peroxide. *Biochim. Biophys. Acta* **1758**, 994–1003 (2006). 16566894 10.1016/j.bbamem.2006.02.015

[30] Henzler, T. & Steudle, E. Transport and metabolic degradation of hydrogen peroxide in Chara corallina: model calculations and measurements with the pressure probe suggest transport of H(2)O(2) across water channels. *J. Exp. Bot.* **51**, 2053–2066 (2000). 11141179 10.1093/jexbot/51.353.2053

[31] García-Dorado, D., Rodríguez-Sinovas, A. & Ruiz-Meana, M. Gap junction-mediated spread of cell injury and death during myocardial ischemia-reperfusion. *Cardiovasc Res.* **61**, 386–401 (2004). 14962471 10.1016/j.cardiores.2003.11.039

[32] Kaya, A., Lee, B. & Gladyshev, V. Regulation of protein function by reversible methionine oxidation and the role of selenoprotein MsrB1. *Antioxid. Redox Signal* **23**, 814–822 (2015). 26181576 10.1089/ars.2015.6385 PMC4589106

[33] Sun, X. et al. The Natural Diterpenoid Isoforretin A Inhibits Thioredoxin-1 and Triggers Potent ROS-Mediated Antitumor Effects. *Cancer Res.* **77**, 926–936 (2017). 10.1158/0008-5472.CAN-16-0987 28011619

[34] Holmström, K. & Finkel, T. Cellular mechanisms and physiological consequences of redox-dependent signalling. *Nat. Rev. Mol. Cell Biol.* **15**, 411–421 (2014). 24854789 10.1038/nrm3801

[35] Dickinson, B. & Chang, C. Chemistry and biology of reactive oxygen species in signaling or stress responses. *Nat. Chem. Biol.* **7**, 504–511 (2011). 21769097 10.1038/nchembio.607 PMC3390228

[36] Bedard, K. & Krause, K. The NOX family of ROS-generating NADPH oxidases: physiology and pathophysiology. *Physiol. Rev.* **87**, 245–313 (2007). 17237347 10.1152/physrev.00044.2005

[37] Parascandolo, A. & Laukkanen, M. Carcinogenesis and Reactive Oxygen Species Signaling: Interaction of the NADPH Oxidase NOX1-5 and Superoxide Dismutase 1-3 Signal Transduction Pathways. *Antioxid. Redox Signal* **30**, 443–486 (2019). 29478325 10.1089/ars.2017.7268 PMC6393772

[38] Yoshihara, A. et al. Regulation of dual oxidase expression and H2O2 production by thyroglobulin. *Thyroid* **22**, 1054–1062 (2012). 22874065 10.1089/thy.2012.0003 PMC3462396

[39] Murphy, M. How mitochondria produce reactive oxygen species. *Biochem. J.* **417**, 1–13 (2009). 19061483 10.1042/BJ20081386 PMC2605959

[40] Boveris, A., Oshino, N. & Chance, B. The cellular production of hydrogen peroxide. *Biochem. J.* **128**, 617–630 (1972). 4404507 10.1042/bj1280617 PMC1173814

[41] Hunt, M., Torres, M., Bachar-Wikström, E. & Wikström, J. Multifaceted roles of mitochondria in wound healing and chronic wound pathogenesis. *Front. Cell Dev. Biol.* **11**, 1252318 (2023). 10.3389/fcell.2023.1252318 PMC10523588 37771375

[42] Wong, H., Benoit, B. & Brand, M. Mitochondrial and cytosolic sources of hydrogen peroxide in resting C2C12 myoblasts. *Free Radic. Biol. Med* **130**, 140–150 (2019). 30389498 10.1016/j.freeradbiomed.2018.10.448

[43] Niedzwiecki, M. et al. The Exposome: Molecules to Populations. *Annu Rev. Pharm. Toxicol.* **59**, 107–127 (2019). 10.1146/annurev-pharmtox-010818-021315 30095351

[44] Rada, B. & Leto, T. Oxidative innate immune defenses by Nox/Duox Family NADPH oxidases. *Contrib. Microbiol* **15**, 164–187 (2008). 18511861 10.1159/000136357 PMC2776633

[45] Martínez-Revelles, S. et al. Reciprocal relationship between reactive oxygen species and cyclooxygenase-2 and vascular dysfunction in hypertension. *Antioxid. Redox Signal* **18**, 51–65 (2013). 22671943 10.1089/ars.2011.4335

[46] Ding, L. et al. Peroxisomal β-oxidation acts as a sensor for intracellular fatty acids and regulates lipolysis. *Nat. Metab.* **3**, 1648–1661 (2021). 34903883 10.1038/s42255-021-00489-2 PMC8688145

[47] Rhee, S. & Kil, I. Multiple Functions and Regulation of Mammalian Peroxiredoxins. *Annu. Rev. Biochem.* **86**, 749–775 (2017). 28226215 10.1146/annurev-biochem-060815-014431

[48] Chance, B., Sies, H. & Boveris, A. Hydroperoxide metabolism in mammalian organs. *Physiol. Rev.* **59**, 527–605 (1979). 37532 10.1152/physrev.1979.59.3.527

[49] Brigelius-Flohé, R. & Flohé, L. Regulatory Phenomena in the Glutathione Peroxidase Superfamily. *Antioxid. Redox Signal* **33**, 498–516 (2020). 31822117 10.1089/ars.2019.7905

[50] Rampon, C., Volovitch, M., Joliot, A. & Vriz, S. Hydrogen Peroxide and Redox Regulation of Developments. *Antioxidants* **7**, 159 (2018). 10.3390/antiox7110159 PMC6262372 30404180

[51] Ronchi, J., Francisco, A., Passos, L., Figueira, T. & Castilho, R. The Contribution of Nicotinamide Nucleotide Transhydrogenase to Peroxide Detoxification Is Dependent on the Respiratory State and Counterbalanced by Other Sources of NADPH in Liver Mitochondria. *J. Biol. Chem.* **291**, 20173–20187 (2016). 27474736 10.1074/jbc.M116.730473 PMC5025700

[52] Hong, W. et al. The Role of Hypoxia-Inducible Factor in Wound Healing. *Adv. Wound Care* **3**, 390–399 (2014). 10.1089/wound.2013.0520 PMC4005494 24804159

[53] Fujiwara, T. et al. Extracellular superoxide dismutase deficiency impairs wound healing in advanced age by reducing neovascularization and fibroblast function. *Exp. Dermatol.* **25**, 206–211 (2016). 26663425 10.1111/exd.12909 PMC4998179

[54] Akhigbe, R. & Ajayi, A. The impact of reactive oxygen species in the development of cardiometabolic disorders: a review. *Lipids Health Dis.* **20**, 23 (2021). 10.1186/s12944-021-01435-7 PMC7916299 33639960

[55] Madreiter-Sokolowski, C., Thomas, C. & Ristow, M. Interrelation between ROS and Ca 2+ in aging and age-related diseases. *Redox Biol*. **36**, 101678 (2020). 10.1016/j.redox.2020.101678 PMC7451758 32810740

[56] Schapira, A. et al. Mitochondrial complex I deficiency in Parkinson’s disease. *J. Neurochem* **54**, 823–827 (1990). 2154550 10.1111/j.1471-4159.1990.tb02325.x

[57] Hasani, M. et al. Oxidative balance score and risk of cancer: a systematic review and meta-analysis of observational studies. *BMC Cancer* **23**, 1143 (2023). 10.1186/s12885-023-11657-w PMC10675899 38001409

[58] Wlaschek, M., Singh, K., Sindrilaru, A., Crisan, D. & Scharffetter-Kochanek, K. Iron and iron-dependent reactive oxygen species in the regulation of macrophages and fibroblasts in non-healing chronic wounds. *Free Radic. Biol. Med.* **133**, 262–275 (2019). 30261274 10.1016/j.freeradbiomed.2018.09.036

[59] Sivaraj, D. et al. Nitric oxide-releasing gel accelerates healing in a diabetic murine splinted excisional wound model. *Front. Med.* **10**, 1060758 (2023). 10.3389/fmed.2023.1060758 PMC10045479 36999070

[60] Papapetropoulos, A., García-Cardeña, G., Madri, J. & Sessa, W. Nitric oxide production contributes to the angiogenic properties of vascular endothelial growth factor in human endothelial cells. *J. Clin. Invest.* **100**, 3131–3139 (1997). 9399960 10.1172/JCI119868 PMC508526

[61] Paul-Clark, M., Gilroy, D., Willis, D., Willoughby, D. & Tomlinson, A. Nitric oxide synthase inhibitors have opposite effects on acute inflammation depending on their route of administration. *J. Immunol.* **166**, 1169–1177 (2001). 11145698 10.4049/jimmunol.166.2.1169

[62] Donnini, S. & Ziche, M. Constitutive and inducible nitric oxide synthase: role in angiogenesis. *Antioxid. Redox Signal* **4**, 817–823 (2002). 12470510 10.1089/152308602760598972

[63] Kitano, T. et al. Impaired Healing of a Cutaneous Wound in an Inducible Nitric Oxide Synthase-Knockout Mouse. *Dermatol. Res. Pr.* **2017**, 2184040 (2017). 10.1155/2017/2184040 PMC5406723 28487726

[64] Yang, Y. et al. Ubiquitination Flow Repressors: Enhancing Wound Healing of Infectious Diabetic Ulcers through Stabilization of Polyubiquitinated Hypoxia-Inducible Factor-1α by Theranostic Nitric Oxide Nanogenerators. *Adv. Mater.* **33**, 2103593 (2021). 10.1002/adma.202103593 34553427

[65] Lin, Y. et al. In Situ Self-Assembling Micellar Depots that Can Actively Trap and Passively Release NO with Long-Lasting Activity to Reverse Osteoporosis. *Adv. Mater*. **30**, e1705605 (2018). 10.1002/adma.201705605 29665153

[66] Radziwon-Balicka, A. et al. Differential eNOS-signalling by platelet subpopulations regulates adhesion and aggregation. *Cardiovasc Res.* **113**, 1719–1731 (2017). 29016749 10.1093/cvr/cvx179 PMC5852541

[67] Arany, I., Brysk, M., Brysk, H. & Tyring, S. Regulation of inducible nitric oxide synthase mRNA levels by differentiation and cytokines in human keratinocytes. *Biochem. Biophys. Res. Commun.* **220**, 618–622 (1996). 8607813 10.1006/bbrc.1996.0452

[68] Krischel, V. et al. Biphasic effect of exogenous nitric oxide on proliferation and differentiation in skin derived keratinocytes but not fibroblasts. *J. Invest. Dermatol.* **111**, 286–291 (1998). 9699731 10.1046/j.1523-1747.1998.00268.x

[69] Stallmeyer, B., Kämpfer, H., Kolb, N., Pfeilschifter, J. & Frank, S. The function of nitric oxide in wound repair: inhibition of inducible nitric oxide-synthase severely impairs wound reepithelialization. *J. Invest. Dermatol.* **113**, 1090–1098 (1999). 10594757 10.1046/j.1523-1747.1999.00784.x

[70] Malone-Povolny, M., Maloney, S. & Schoenfisch, M. Nitric Oxide Therapy for Diabetic Wound Healing. *Adv. Health. Mater.* **8**, e1801210 (2019). 10.1002/adhm.201801210 PMC6774257 30645055

[71] Peters, S., Mathy, M., Pfaffendorf, M. & van Zwieten, P. Reactive oxygen species-induced aortic vasoconstriction and deterioration of functional integrity. *Naunyn Schmiedebergs Arch. Pharm.* **361**, 127–133 (2000). 10.1007/s002109900148 10685867

[72] Razzell, W., Evans, I., Martin, P. & Wood, W. Calcium flashes orchestrate the wound inflammatory response through DUOX activation and hydrogen peroxide release. *Curr. Biol.* **23**, 424–429 (2013). 23394834 10.1016/j.cub.2013.01.058 PMC3629559

[73] de Oliveira, S. et al. ATP modulates acute inflammation in vivo through dual oxidase 1-derived H2O2 production and NF-κB activation. *J. Immunol.* **192**, 5710–5719 (2014). 24842759 10.4049/jimmunol.1302902

[74] Eming, S., Wynn, T. & Martin, P. Inflammation and metabolism in tissue repair and regeneration. *Science* **56**, 1026–1030 (2017). 10.1126/science.aam7928 28596335

[75] Weinberg, S., Sena, L. & Chandel, N. Mitochondria in the regulation of innate and adaptive immunity. *Immunity* **42**, 406–417 (2015). 25786173 10.1016/j.immuni.2015.02.002 PMC4365295

[76] Willenborg, S. et al. Mitochondrial metabolism coordinates stage-specific repair processes in macrophages during wound healing. *Cell Metab.* **33**, 2398–2414.e9 (2020). 10.1016/j.cmet.2021.10.004 34715039

[77] Lee, C., Qiao, M., Schröder, K., Zhao, Q. & Asmis, R. Nox4 is a novel inducible source of reactive oxygen species in monocytes and macrophages and mediates oxidized low density lipoprotein-induced macrophage death. *Circ. Res.* **106**, 1489–1497 (2010). 20360249 10.1161/CIRCRESAHA.109.215392 PMC2924578

[78] Kim, S. & Nair, M. Macrophages in wound healing: activation and plasticity. *Immunol. Cell Biol.* **97**, 258–267 (2019). 30746824 10.1111/imcb.12236 PMC6426672

[79] Bulua, A. et al. Mitochondrial reactive oxygen species promote production of proinflammatory cytokines and are elevated in TNFR1-associated periodic syndrome (TRAPS). *J. Exp. Med.* **208**, 519–533 (2011). 21282379 10.1084/jem.20102049 PMC3058571

[80] Zhao, C., Gillette, D., Li, X., Zhang, Z. & Wen, H. Nuclear factor E2-related factor-2 (Nrf2) is required for NLRP3 and AIM2 inflammasome activation. *J. Biol. Chem.* **289**, 17020–17029 (2014). 24798340 10.1074/jbc.M114.563114 PMC4059144

[81] Cuadrado, A. et al. Transcription Factor NRF2 as a Therapeutic Target for Chronic Diseases: A Systems Medicine Approach. *Pharm. Rev.* **70**, 348–383 (2018). 29507103 10.1124/pr.117.014753

[82] Bonello, S. et al. Reactive oxygen species activate the HIF-1alpha promoter via a functional NFkappaB site. *Arterioscler Thromb. Vasc. Biol.* **27**, 755–761 (2007). 17272744 10.1161/01.ATV.0000258979.92828.bc

[83] Ranneh, Y. et al. Crosstalk between reactive oxygen species and pro-inflammatory markers in developing various chronic diseases: a review. *Appl Biol. Chem.* **60**, 327–338 (2017).

[84] Villarino, A., Kanno, Y. & O’Shea, J. Mechanisms and consequences of Jak-STAT signaling in the immune system. *Nat. Immunol*., **18**, 374-384 (2017). 10.1038/ni.3691 PMC11565648 28323260

[85] Ostadkarampour, M. & Putnins, E. Monoamine Oxidase Inhibitors: A Review of Their Anti-Inflammatory Therapeutic Potential and Mechanisms of Action. *Front. Pharm.* **12**, 676239 (2021). 10.3389/fphar.2021.676239 PMC8120032 33995107

[86] Bhattacharjee, A. et al. IL-4 and IL-13 employ discrete signaling pathways for target gene expression in alternatively activated monocytes/macrophages. *Free Radic. Biol. Med.* **54**, 1–16 (2013). 23124025 10.1016/j.freeradbiomed.2012.10.553 PMC3534796

[87] Ekuni, D. et al. Lipopolysaccharide-induced epithelial monoamine oxidase mediates alveolar bone loss in a rat chronic wound model. *Am. J. Pathol.* **175**, 1398–1409 (2009). 19779138 10.2353/ajpath.2009.090108 PMC2751537

[88] Fogarty, C. et al. Extracellular Reactive Oxygen Species Drive Apoptosis-Induced Proliferation via Drosophila Macrophages. *Curr. Biol.* **26**, 575–584 (2016). 26898463 10.1016/j.cub.2015.12.064 PMC4765900

[89] Canton, M. et al. Reactive Oxygen Species in Macrophages: Sources and Targets. *Front. Immunol.* **12**, 734229 (2021). 34659222 10.3389/fimmu.2021.734229 PMC8515906

[90] Bian, Z. et al. Deciphering human macrophage development at single-cell resolution. *Nature* **582**, 571–576 (2020). 32499656 10.1038/s41586-020-2316-7

[91] Zheng, H. et al. Recent advances in strategies to target the behavior of macrophages in wound healing. *Biomed. Pharmacother.* **165**, 115199 (2023). 37517288 10.1016/j.biopha.2023.115199

[92] Xiao, Y. et al. Single-cell profiling and functional screening reveal crucial roles for lncRNAs in the epidermal re-epithelialization of human acute wounds. *Front. Surg.* **11**, 1349135 (2024). 38468869 10.3389/fsurg.2024.1349135 PMC10925684

[93] Falanga, V. et al. Chronic wounds. *Nat. Rev. Dis. Prim.* **8**, 50 (2022). 35864102 10.1038/s41572-022-00377-3 PMC10352385

[94] Clark, R. Basics of cutaneous wound repair. *J. Dermatol Surg. Oncol.* **19**, 693–706 (1993). 8349909 10.1111/j.1524-4725.1993.tb00413.x

[95] Geiszt, M., Witta, J., Baffi, J., Lekstrom, K. & Leto, T. Dual oxidases represent novel hydrogen peroxide sources supporting mucosal surface host defense. *FASEB J.* **17**, 1502–1504 (2003). 12824283 10.1096/fj.02-1104fje

[96] Ha, E., Oh, C., Bae, Y. & Lee, W. A direct role for dual oxidase in Drosophila gut immunity. *Science* **310**, 847–850 (2005). 16272120 10.1126/science.1117311

[97] Cavallo, I. et al. Homocysteine and Inflammatory Cytokines in the Clinical Assessment of Infection in Venous Leg Ulcers. *Antibiotics* **11**, 1268 (2022). 10.3390/antibiotics11091268 PMC9495878 36140047

[98] Winterbourn, C., Kettle, A. & Hampton, M. Reactive Oxygen Species and Neutrophil Function. *Annu. Rev. Biochem.* **85**, 765–792 (2016). 27050287 10.1146/annurev-biochem-060815-014442

[99] Kenny, E. et al. Diverse stimuli engage different neutrophil extracellular trap pathways. *Elife* **6**, e24437 (2017). 28574339 10.7554/eLife.24437 PMC5496738

[100] Pase, L., Nowell, C. & Lieschke, G. In vivo real-time visualization of leukocytes and intracellular hydrogen peroxide levels during a zebrafish acute inflammation assay. *Methods Enzymol.* **506**, 135–156 (2012). 22341223 10.1016/B978-0-12-391856-7.00032-9

[101] Ojha, N. et al. Assessment of wound-site redox environment and the significance of Rac2 in cutaneous healing. *Free Radic. Biol. Med.* **44**, 682–691 (2008). 18068132 10.1016/j.freeradbiomed.2007.10.056 PMC2719562

[102] Méndez, D. et al. Mitoquinone (MitoQ) Inhibits Platelet Activation Steps by Reducing ROS Levels. *Int. J. Mol. Sci.* **21**, 6192 (2020). 32867213 10.3390/ijms21176192 PMC7503844

[103] Fidler, T. et al. Superoxide Dismutase 2 is dispensable for platelet function. *Thromb. Haemost.* **117**, 1859–1867 (2017). 28771279 10.1160/TH17-03-0174 PMC5894334

[104] Kim, S. et al. Platelet-derived mitochondria transfer facilitates wound-closure by modulating ROS levels in dermal fibroblasts. *Platelets* **34**, 2151996 (2022). 10.1080/09537104.2022.2151996 36529914

[105] Li, W., Liu, G., Chou, I. & Kagan, H. Hydrogen peroxide-mediated, lysyl oxidase-dependent chemotaxis of vascular smooth muscle cells. *J. Cell Biochem* **78**, 550–557 (2000). 10861852

[106] Brüne, B. Nitric oxide: NO apoptosis or turning it ON? *Cell Death Differ.* **10**, 864–869 (2003). 12867993 10.1038/sj.cdd.4401261

[107] Cai, Z. et al. Endothelial Nitric Oxide Synthase-Derived Nitric Oxide Prevents Dihydrofolate Reductase Degradation via Promoting S-Nitrosylation. *Arterioscler Thromb. Vasc. Biol.* **35**, 2366–2373 (2015). 26381869 10.1161/ATVBAHA.115.305796 PMC4758687

[108] Wang, J., Hong, Z., Zeng, C., Yu, Q. & Wang, H. NADPH oxidase 4 promotes cardiac microvascular angiogenesis after hypoxia/reoxygenation in vitro *Free*. *Radic. Biol. Med*. **69**, 278–288 (2014). 10.1016/j.freeradbiomed.2014.01.027 24480752

[109] Martinotti, S., Patrone, M., Balbo, V., Mazzucco, L. & Ranzato, E. Endothelial response boosted by platelet lysate: the involvement of calcium toolkit. *Int. J. Mol. Sci*. **21**, 808 (2020). 10.3390/ijms21030808 PMC7036775 31991927

[110] Evangelista, A., Thompson, M., Bolotina, V., Tong, X. & Cohen, R. Nox4- and Nox2-dependent oxidant production is required for VEGF-induced SERCA cysteine-674 S-glutathiolation and endothelial cell migration. *Free Radic. Biol. Med.* **53**, 2327–2334 (2012). 23089226 10.1016/j.freeradbiomed.2012.10.546 PMC3568680

[111] Yang, J. The role of reactive oxygen species in angiogenesis and preventing tissue injury after brain ischemia. *Microvasc. Res.* **123**, 62–67 (2019). 30594490 10.1016/j.mvr.2018.12.005

[112] Menden, H., Welak, S., Cossette, S., Ramchandran, R. & Sampath, V. Lipopolysaccharide (LPS)-mediated angiopoietin-2-dependent autocrine angiogenesis is regulated by NADPH oxidase 2 (Nox2) in human pulmonary microvascular endothelial cells. *J. Biol. Chem.* **290**, 5449–5461 (2015). 25568324 10.1074/jbc.M114.600692 PMC4342461

[113] Brown, N. & Bicknell, R. Thymidine phosphorylase, 2-deoxy-D-ribose and angiogenesis. *Biochem J.* **334**, 1–8 (1998). 9693094 10.1042/bj3340001 PMC1219653

[114] Go, Y. & Jones, D. The redox proteome. *J. Biol. Chem.* **288**, 26512–26520 (2013). 23861437 10.1074/jbc.R113.464131 PMC3772199

[115] Pak, V. et al. Ultrasensitive Genetically Encoded Indicator for Hydrogen Peroxide Identifies Roles for the Oxidant in Cell Migration and Mitochondrial Function. *Cell Metab.* **31**, 642–653.e646 (2020). 32130885 10.1016/j.cmet.2020.02.003 PMC7088435

[116] Balta, E., Kramer, J. & Samstag, Y. Redox Regulation of the Actin Cytoskeleton in Cell Migration and Adhesion: On the Way to a Spatiotemporal View. *Front. Cell Dev. Biol.* **8**, 618261 (2021). 33585453 10.3389/fcell.2020.618261 PMC7875868

[117] Hunter, M., Willoughby, P., AEE, B. & Fernandez-Gonzalez, R. Oxidative Stress Orchestrates Cell Polarity to Promote Embryonic Wound Healing. *Dev. Cell* **47**, 377–387 (2018). 30399336 10.1016/j.devcel.2018.10.013

[118] Xu, S. & Chisholm, A. C. elegans epidermal wounding induces a mitochondrial ROS burst that promotes wound repair. *Dev. Cell* **31**, 48–60 (2014). 25313960 10.1016/j.devcel.2014.08.002 PMC4197410

[119] Sherwood, C., Lantz, R., Burgess, J. & Boitano, S. Arsenic alters ATP-dependent Ca²+ signaling in human airway epithelial cell wound response. *Toxicol. Sci.* **121**, 191–206 (2011). 21357385 10.1093/toxsci/kfr044 PMC3080191

[120] Bucheimer, R. & Linden, J. Purinergic regulation of epithelial transport. *J. Physiol.* **555**, 311–321 (2004). 14694149 10.1113/jphysiol.2003.056697 PMC1664845

[121] Pillai, S. & Bikle, D. Adenosine triphosphate stimulates phosphoinositide metabolism, mobilizes intracellular calcium, and inhibits terminal differentiation of human epidermal keratinocytes. *J. Clin. Invest.* **90**, 42–51 (1992). 1321844 10.1172/JCI115854 PMC443061

[122] Van Huizen, A., Hack, S., Greene, J., Kinsey, L. & Beane, W. Reactive Oxygen Species Signaling Differentially Controls Wound Healing and Regeneration. *bioRxiv*, https://www.biorxiv.org/content/10.1101/2022.04.05.487111v1.full (2022).

[123] Yue, C. et al. c-Jun Overexpression Accelerates Wound Healing in Diabetic Rats by Human Umbilical Cord-Derived Mesenchymal Stem Cells. *Stem Cells Int.* **2020**, 7430968 (2020). 10.1155/2020/7430968 PMC7201444 32399050

[124] Fujino, G. et al. Thioredoxin and TRAF family proteins regulate reactive oxygen species-dependent activation of ASK1 through reciprocal modulation of the N-terminal homophilic interaction of ASK1. *Mol. Cell Biol.* **27**, 8152–8163 (2007). 17724081 10.1128/MCB.00227-07 PMC2169188

[125] Esteban-Collado, J., Corominas, M. & Serras, F. Nutrition and PI3K/Akt signaling are required for p38-dependent regeneration. *Development* **148**, dev197087 (2021). 10.1242/dev.197087 33913483

[126] Patel, P. et al. Damage sensing by a Nox-Ask1-MKK3-p38 signaling pathway mediates regeneration in the adult Drosophila midgut. *Nat. Commun.* **10**, 4365 (2019). 31554796 10.1038/s41467-019-12336-w PMC6761285

[127] Manchanda, M. et al. Metabolic Reprogramming and Reliance in Human Skin Wound Healing. *J. Invest Dermatol.* **S0022-202X**, 01975–01979 (2023). 10.1016/j.jid.2023.02.039 37061123

[128] Romero, M., McCathie, G., Jankun, P. & Roehl, H. Damage-induced reactive oxygen species enable zebrafish tail regeneration by repositioning of Hedgehog expressing cells. *Nat. Commun.* **9**, 4010 (2018). 30275454 10.1038/s41467-018-06460-2 PMC6167316

[129] Meda, F. et al. Nerves Control Redox Levels in Mature Tissues Through Schwann Cells and Hedgehog Signaling. *Antioxid. Redox Signal* **24**, 299–311 (2016). 26442784 10.1089/ars.2015.6380 PMC4761803

[130] Thauvin, M. et al. An early Shh-H2O2 reciprocal regulatory interaction controls the regenerative program during zebrafish fin regeneration. *J. Cell Sci.* **135**, jcs259664 (2022). 35107164 10.1242/jcs.259664

[131] Singh, B., Doyle, M., Weaver, C., Koyano-Nakagawa, N. & Garry, D. Hedgehog and Wnt coordinate signaling in myogenic progenitors and regulate limb regeneration. *Dev. Biol.* **371**, 23–34 (2012). 22902898 10.1016/j.ydbio.2012.07.033 PMC3987681

[132] Hamanaka, R. et al. Mitochondrial reactive oxygen species promote epidermal differentiation and hair follicle development. *Sci Signal* **6**, ra8 (2013). 10.1126/scisignal.2003638 PMC4017376 23386745

[133] LeBert, D. et al. Damage-induced reactive oxygen species regulate v*imentin* and dynamic collagen-based projections to mediate wound repair. *Elife* **7**, e30703 (2018). 10.7554/eLife.30703 PMC5790375 29336778

[134] Morgan, M. & Liu, Z. Crosstalk of reactive oxygen species and NF-kappaB signaling. *Cell Res.* **21**, 103–115 (2011). 21187859 10.1038/cr.2010.178 PMC3193400

[135] Kamata, H., Manabe, T., Oka, S., Kamata, K. & Hirata, H. Hydrogen peroxide activates IkappaB kinases through phosphorylation of serine residues in the activation loops. *FEBS Lett.* **519**, 231–237 (2002). 12023051 10.1016/s0014-5793(02)02712-6

[136] Halvey, P. et al. Selective oxidative stress in cell nuclei by nuclear-targeted D-amino acid oxidase. *Antioxid. Redox Signal* **9**, 807–816 (2007). 17508907 10.1089/ars.2007.1526

[137] Schwörer, S. et al. Proline biosynthesis is a vent for TGFβ-induced mitochondrial redox stress. *EMBO J.* **39**, e103334 (2020). 32134147 10.15252/embj.2019103334 PMC7156964

[138] Bansal, R. et al. Role of the mitochondrial protein cyclophilin D in skin wound healing and collagen secretion. *JCI Insight* **9**, e169213 (2024). 38564292 10.1172/jci.insight.169213 PMC11141914

[139] Zhao, F., Wei, W., Huang, D. & Guo, Y. Knockdown of miR-27a reduces TGFβ-induced EMT and H 2 O 2 -induced oxidative stress through regulating mitochondrial autophagy. *Am. J. Transl. Res.* **15**, 6071–6082 (2023). 37969181 PMC10641347

[140] Yoo, S., Freisinger, C., LeBert, D. & Huttenlocher, A. Early redox, Src family kinase, and calcium signaling integrate wound responses and tissue regeneration in zebrafish. *J. Cell Biol.* **199**, 225–234 (2012). 23045550 10.1083/jcb.201203154 PMC3471241

[141] Cabodi, S. et al. A PKC-eta/Fyn-dependent pathway leading to keratinocyte growth arrest and differentiation. *Mol. Cell* **6**, 1121–1129 (2000). 11106751 10.1016/s1097-2765(00)00110-6

[142] Saito, Y., Jensen, A., Salgia, R. & Posadas, E. Fyn: a novel molecular target in cancer. *Cancer* **116**, 1629–1637 (2010). 20151426 10.1002/cncr.24879 PMC2847065

[143] Klotz, L. & Steinbrenner, H. Cellular adaptation to xenobiotics: Interplay between xenosensors, reactive oxygen species and FOXO transcription factors. *Redox Biol.* **13**, 646–654 (2017). 28818793 10.1016/j.redox.2017.07.015 PMC5558470

[144] Miao, C., Li, Y. & Zhang, X. The functions of FoxO transcription factors in epithelial wound healing. *Australas. J. Dermatol* **60**, 105–109 (2019). 30450624 10.1111/ajd.12952

[145] Jeon, H. et al. FOXO1 regulates VEGFA expression and promotes angiogenesis in healing wounds. *J. Pathol.* **245**, 258–264 (2018). 29574902 10.1002/path.5075 PMC6566901

[146] Ponugoti, B. et al. FOXO1 promotes wound healing through the up-regulation of TGF-β1 and prevention of oxidative stress. *J. Cell Biol.* **203**, 327–343 (2013). 24145170 10.1083/jcb.201305074 PMC3812981

[147] Dhoke, N., Geesala, R. & Das, A. Low Oxidative Stress-Mediated Proliferation V*ia* JNK-FOXO3a-Catalase Signaling in Transplanted Adult Stem Cells Promotes Wound Tissue Regeneration. *Antioxid. Redox Signal* **28**, 1047–1065 (2018). 28826225 10.1089/ars.2016.6974

[148] Tonelli, C., Chio, I. & Tuveson, D. Transcriptional Regulation by Nrf2. *Antioxid. Redox Signal* **29**, 1727–1745 (2018). 28899199 10.1089/ars.2017.7342 PMC6208165

[149] Schmidt, A. & Bekeschus, S. Redox for Repair: Cold Physical Plasmas and Nrf2 Signaling Promoting Wound Healing. *Antioxidants* **7**, 146 (2018). 10.3390/antiox7100146 PMC6210784 30347767

[150] Fourquet, S., Guerois, R., Biard, D. & Toledano, M. Activation of NRF2 by nitrosative agents and H2O2 involves KEAP1 disulfide formation. *J. Biol. Chem.* **285**, 8463–8471 (2010). 20061377 10.1074/jbc.M109.051714 PMC2832995

[151] Süntar, I. et al. Regulatory Role of Nrf2 Signaling Pathway in Wound Healing Process. *Molecules* **26**, 2424 (2021). 33919399 10.3390/molecules26092424 PMC8122529

[152] Long, M. et al. An Essential Role of NRF2 in Diabetic Wound Healing. *Diabetes* **65**, 780–793 (2016). 26718502 10.2337/db15-0564 PMC4764153

[153] Kuhn, J. et al. Nrf2-activating Therapy Accelerates Wound Healing in a Model of Cutaneous Chronic Venous Insufficiency. *Plast. Reconstr. Surg. Glob. Open* **8**, e3006 (2020). 33299679 10.1097/GOX.0000000000003006 PMC7722614

[154] Li, D. et al. LPS-stimulated Macrophage Exosomes Inhibit Inflammation by Activating the Nrf2 / HO-1 Defense Pathway and Promote Wound Healing in Diabetic Rats, 2020, PREPRINT. Res. Sq. Preprint at: 10.21203/rs.3.rs-78864/v1 (2020).

[155] Fan, J. et al. Procyanidin B2 improves endothelial progenitor cell function and promotes wound healing in diabetic mice via activating Nrf2. *J. Cell Mol. Med*. **25**, 652–665 (2021). 33215883 10.1111/jcmm.16111 PMC7812287

[156] Li, M. et al. Nrf2 Suppression Delays Diabetic Wound Healing Through Sustained Oxidative Stress and Inflammation. *Front Pharm.* **10**, 1099 (2019). 10.3389/fphar.2019.01099 PMC6763603 31616304

[157] Hozzein, W. et al. Bee venom improves diabetic wound healing by protecting functional macrophages from apoptosis and enhancing Nrf2, Ang-1 and Tie-2 signaling. *Mol. Immunol.* **103**, 322–335 (2018). 30366166 10.1016/j.molimm.2018.10.016

[158] Rieger, S. & Sagasti, A. Hydrogen peroxide promotes injury-induced peripheral sensory axon regeneration in the zebrafish skin. *PLoS Biol.* **9** e1000621 (2011). 10.1371/journal.pbio.1000621 PMC3101194 21629674

[159] Schäfer, M. & Werner, S. Oxidative stress in normal and impaired wound repair. *Pharm. Res.* **58**, 165–171 (2008). 10.1016/j.phrs.2008.06.004 18617006

[160] Lord, M. et al. Perlecan and vascular endothelial growth factor-encoding DNA-loaded chitosan scaffolds promote angiogenesis and wound healing. *J. Control Rel.* **250**, 48–61 (2017). 10.1016/j.jconrel.2017.02.009 PMC9727826 28189628

[161] Seiwerth, S. et al. BPC 157 and Standard Angiogenic Growth Factors. Gastrointestinal Tract Healing, Lessons from Tendon, Ligament, Muscle and Bone Healing. *Curr. Pharm. Des.* **24**, 1972–1989 (2018). 29998800 10.2174/1381612824666180712110447

[162] Moseley, R., Stewart, J., Stephens, P., Waddington, R. & Thomas, D. Extracellular matrix metabolites as potential biomarkers of disease activity in wound fluid: lessons learned from other inflammatory diseases? *Br. J. Dermatol.* **150**, 401–413 (2004). 15030321 10.1111/j.1365-2133.2004.05845.x

[163] Perillo, B. et al. ROS in cancer therapy: the bright side of the moon. *Exp. Mol. Med.* **52**, 192–203 (2020). 32060354 10.1038/s12276-020-0384-2 PMC7062874

[164] Chasara, R., Ajayi, T., Leshilo, D., Poka, M. & Witika, B. Exploring novel strategies to improve anti-tumour efficiency: The potential for targeting reactive oxygen species. *Heliyon* **9**, e19896 (2023). 10.1016/j.heliyon.2023.e19896 PMC10559285 37809420

[165] Aborode, A. et al. Targeting Oxidative Stress Mechanisms to Treat Alzheimer’s and Parkinson’s Disease: A Critical Review. *Oxidative Med. Cell Longev.* **2022**, 7934442 (2022). 10.1155/2022/7934442 PMC9357807 35958022

[166] Chávez, M. & Tse, H. Targeting Mitochondrial-Derived Reactive Oxygen Species in T Cell-Mediated Autoimmune Diseases. *Front. Immunol.* **12**, 703972 (2021). 34276700 10.3389/fimmu.2021.703972 PMC8281042

[167] Khan, A. et al. Targeting deregulated oxidative stress in skin inflammatory diseases: An update on clinical importance. *Biomed. Pharmacother.* **154**, 113601 (2022). 36049315 10.1016/j.biopha.2022.113601

[168] Wilkinson, H. & Hardman, M. Senescence in Wound Repair: Emerging Strategies to Target Chronic Healing Wounds. *Front. Cell Dev. Biol.* **11**, 773 (2020). **8**. 10.3389/fcell.2020.00773 PMC7431694 32850866

[169] Duan, J., Duan, J., Zhang, Z. & Tong, T. Irreversible cellular senescence induced by prolonged exposure to H2O2 involves DNA-damage-and-repair genes and telomere shortening. *Int. J. Biochem. Cell Biol.* **37**, 1407–1420 (2005). 15833273 10.1016/j.biocel.2005.01.010

[170] Ruan, Y., Wu, S., Zhang, L., Chen, G. & Lai, W. Retarding the senescence of human vascular endothelial cells induced by hydrogen peroxide: effects of 17beta-estradiol (E2) mediated mitochondria protection. *Biogerontology* **15**, 367–375 (2014). 24938685 10.1007/s10522-014-9507-2

[171] Herrling, T., Jung, K. & Fuchs, J. Measurements of UV-generated free radicals/reactive oxygen species (ROS) in skin. *Spectrochim. Acta A Mol. Biomol. Spectrosc.* **63**, 840–845 (2006). 16543118 10.1016/j.saa.2005.10.013

[172] Buranasin, P. et al. High glucose-induced oxidative stress impairs proliferation and migration of human gingival fibroblasts. *PLoS ONE* **13**, e0201855 (2018). 30092096 10.1371/journal.pone.0201855 PMC6084939

[173] Arul, V. et al. Glucose Oxidase Incorporated Collagen Matrices for Dermal Wound Repair in Diabetic Rat Models: A Biochemical Study. *J. Biomater. Appl* **26**, 917–938 (2012). 21363874 10.1177/0885328210390402

[174] Zhang, L. et al. A composite hydrogel of chitosan/heparin/poly (γ-glutamic acid) loaded with superoxide dismutase for wound healing. *Carbohydr. Polym.* **180**, 168–174 (2018). 29103492 10.1016/j.carbpol.2017.10.036

[175] Beer, H., Longaker, M. & Werner, S. Reduced Expression of PDGF and PDGF Receptors During Impaired Wound Healing. *J. Investig. Dermatol* **109**, 132–138 (1997). 9242497 10.1111/1523-1747.ep12319188

[176] Kaltalioglu, K., Coskun-Cevher, S., Tugcu-Demiroz, F. & Celebi, N. PDGF supplementation alters oxidative events in wound healing process: a time course study. *Arch. Dermatol Res.* **305**, 415–422 (2013). 23423159 10.1007/s00403-013-1326-9

[177] Lin, Y. et al. Galectin-1 Accelerates Wound Healing by Regulating the Neuropilin-1/Smad3/NOX4 Pathway and ROS Production in Myofibroblasts. *J. Investig. Dermatol.* **135**, 258–268 (2015). 25007042 10.1038/jid.2014.288

[178] Polouliakh, N. et al. Alpha-Arbutin Promotes Wound Healing by Lowering ROS and Upregulating Insulin/IGF-1 Pathway in Human Dermal Fibroblast. *Front. Physiol.* **11**, 586843 (2020). 33250779 10.3389/fphys.2020.586843 PMC7672191

[179] Quiros, M. et al. Resolvin E1 is a pro-repair molecule that promotes intestinal epithelial wound healing. *Proc. Natl Acad. Sci.* **117**, 9477–9482 (2020). 32300016 10.1073/pnas.1921335117 PMC7197018

[180] Chen, S. et al. Topical treatment with anti-oxidants and Au nanoparticles promote healing of diabetic wound through receptor for advance glycation end-products. *Eur. J. Pharm. Sci.* **47**, 875–883 (2012). 22985875 10.1016/j.ejps.2012.08.018

[181] Chigurupati, S. et al. Effects of cerium oxide nanoparticles on the growth of keratinocytes, fibroblasts and vascular endothelial cells in cutaneous wound healing. *Biomaterials* **34**, 2194–2201 (2013). 23266256 10.1016/j.biomaterials.2012.11.061 PMC3552035

[182] Zhang, S. et al. Polydopamine/puerarin nanoparticle-incorporated hybrid hydrogels for enhanced wound healing. *Biomater. Sci.* **7**, 4230–4236 (2019). 31393463 10.1039/c9bm00991d

[183] Liu, T. et al. Ultrasmall copper-based nanoparticles for reactive oxygen species scavenging and alleviation of inflammation related diseases. *Nat. Commun.* **11**, 2788 (2020). 32493916 10.1038/s41467-020-16544-7 PMC7270130

[184] Mao, L. et al. In Situ Synthesized Selenium Nanoparticles‐Decorated Bacterial Cellulose/Gelatin Hydrogel with Enhanced Antibacterial, Antioxidant, and Anti‐Inflammatory Capabilities for Facilitating Skin Wound Healing. *Adv. Health. Mater.* **10**, e2100402 (2021). 10.1002/adhm.202100402 34050616

[185] Kaur, S. et al. Galvanic zinc–copper microparticles produce electrical stimulation that reduces the inflammatory and immune responses in skin. *Arch. Dermatol Res.* **303**, 551 (2011). 21465312 10.1007/s00403-011-1145-9

[186] Tandon, N. et al. Galvanic microparticles increase migration of human dermal fibroblasts in a wound-healing model via reactive oxygen species pathway. *Exp. Cell Res.* **320**, 79–91 (2014). 24113575 10.1016/j.yexcr.2013.09.016 PMC4480867

[187] Sahu, A., Jeon, J., Lee, M., Yang, H. & Tae, G. Antioxidant and anti-inflammatory activities of Prussian blue nanozyme promotes full-thickness skin wound healing. *Mater. Sci. Eng C.* **119**, 111596 (2021). 10.1016/j.msec.2020.111596 33321640

[188] Lee, C. et al. Light-Stimulated Carbon Dot Hydrogel: Targeting and Clearing Infectious Bacteria In Vivo. *ACS Appl. Bio Mater.* **5**, 761–770 (2022). 35020368 10.1021/acsabm.1c01157

[189] Tur, E., Bolton, L. & Constantine, B. Topical hydrogen peroxide treatment of ischemic ulcers in the guinea pig: Blood recruitment in multiple skin sites. *J. Am. Acad. Dermatol* **33**, 217–221 (1995). 7622648 10.1016/0190-9622(95)90238-4

[190] Huang, X. et al. Hyperbaric oxygen potentiates diabetic wound healing by promoting fibroblast cell proliferation and endothelial cell angiogenesis. *Life Sci.* **259**, 118246 (2020). 32791151 10.1016/j.lfs.2020.118246

[191] Teguh, D. et al. Hyperbaric oxygen therapy for nonhealing wounds: Treatment results of a single center. *Wound Repair Regen.* **29**, 254–260 (2021). 33377598 10.1111/wrr.12884 PMC7986203

[192] Capó, X. et al. Hyperbaric Oxygen Therapy Reduces Oxidative Stress and Inflammation, and Increases Growth Factors Favouring the Healing Process of Diabetic Wounds. *Int. J. Mol. Sci.* **24**, 7040 (2023). 37108205 10.3390/ijms24087040 PMC10139175

[193] Zeng, Q., Shi, G. & Zhang, M. Real‐Time Monitoring of Wound States via Rationally Engineered Biosensors. *Adv. Sens. Res.* **3**, 2200018 (2024).

[194] Bachar-Wikstrom, E. et al. Endoplasmic reticulum stress in human chronic wound healing: Rescue by 4-phenylbutyrate. *Int. Wound J.* **18**, 49–61 (2021). 33225583 10.1111/iwj.13525 PMC7949014

[195] Masson-Meyers, D. et al. Experimental models and methods for cutaneous wound healing assessment. *Int. J. Exp. Pathol.* **101**, 21–37 (2020). 32227524 10.1111/iep.12346 PMC7306904

[196] Roy, S., Khanna, S., Nallu, K., Hunt, T. & Sen, C. Dermal wound healing is subject to redox control. *Mol. Ther.* **13**, 211–220 (2006). 16126008 10.1016/j.ymthe.2005.07.684 PMC1389791

[197] Juarez, M., Patterson, R., Sandoval-Guillen, E. & McGinnis, W. Duox, Flotillin-2, and Src42A are required to activate or delimit the spread of the transcriptional response to epidermal wounds in Drosophila. *PLoS Genet* **7**, e1002424 (2011). 22242003 10.1371/journal.pgen.1002424 PMC3248467

[198] Son, D. et al. A Novel Peptide, Nicotinyl–Isoleucine–Valine–Histidine (NA–IVH), Promotes Antioxidant Gene Expression and Wound Healing in HaCaT Cells. *Mar. Drugs* **16**, 262 (2018). 30071627 10.3390/md16080262 PMC6117656

[199] Frykberg, R. et al. Use of Topical Oxygen Therapy in Wound Healing. *J. Wound Care* **32**, S1–S32 (2023). 37607744 10.12968/jowc.2023.32.Sup8b.S1

